# Supplementary material for: Molecular architecture of the yeast Mediator complex
Source: eLife. 2015 Sep 24;4:e08719. doi: 10.7554/eLife.08719 (PMC4631838; doi:10.7554/eLife.08719)
Supplement: Figure 1—source data 1. — Numbers of unique cross-linked residue pairs (‘cross-links’) and cross-linked spectral matches used for integrative modeling of Mediator apo-complex (‘Total Mediator’) as well as total number of holoenzyme cross-links identified in this study (‘Total [this study]’). DOI: http://dx.doi.org/10.7554/eLife.08719.004 [file elife08719s001.docx]

| **Cross-link Class** | **Unique Cross-links** | **Cross-link Spectral Matches** |
| --- | --- | --- |
| Head Module | 52 | 561 |
| Middle Module (this study) | 89 | 770 |
| Middle Module (Larivière et al., 2013) | 34 | 51 |
| Middle Module (common) | 4 | NA |
| Tail Module | 92 | 474 |
| Inter-module Mediator | 23 | 44 |
| **Total Mediator** | **294** | **1900** |
| Polymerase | 124 | 1299 |
| Inter-module Mediator-Polymerase | 18 | 48 |
| **Total (this study)** | **402** | **3196** |

**Figure 1 – source data 1**
