# Supplementary material for: Molecular architecture of the yeast Mediator complex
Source: eLife. 2015 Sep 24;4:e08719. doi: 10.7554/eLife.08719 (PMC4631838; doi:10.7554/eLife.08719)
Supplement: Figure 5—source data 1. — DOI: http://dx.doi.org/10.7554/eLife.08719.016 [file elife08719s002.docx]

| **Query Protein** | **Query Residues** | **Template Protein** | **PDB code (Chain)** | **Template Residues** | **P-value** | **HHpred Score** |
| --- | --- | --- | --- | --- | --- | --- |
| Med2 | 31-156 | Fibrinogen alpha | 1DEQ (A) | 53-180 | 1.1E-05 | 45.3 |
| Med2 | 67-160 | Fibrinogen beta | 1M1J (B) | 92-185 | 3.6E-05 | 45.2 |
| Med2 | 44-151 | Fibrinogen gamma | 1M1J (C) | 26-132 | 2.8E-03 | 30.8 |
| Med3 | 10-210 | Fibrinogen alpha | 1DEQ (A) | 121-326 | 2.6E-04 | 35.9 |
| Med3 | 10-108 | Fibrinogen beta | 1M1J (B) | 101-200 | 1.2E-03 | 34.2 |
| Med3 | 10-97 | Fibrinogen gamma | 1M1J (C) | 39-133 | 2.2E-03 | 31.3 |
| Med15 | 429-590 | Fibrinogen alpha | 3GHG (A) | 39-152 | 1.8E-03 | 36.8 |
| Med16 | 6-538 | Sec31 | 2PM9 (A) | 16-415 | 1.7E-06 | 59.4 |

**Figure 5 – source data 1**
